# Heuristic Vetoing: Top-Down Influences of the Anchoring-and-Adjustment Heuristic Can Override the Bottom-Up Information in Visual Images

**DOI:** 10.3389/fnins.2022.745269

**Published:** 2022-05-20

**Authors:** Fallon Branch, Erin Park, Jay Hegdé

**Affiliations:** ^1^Department of Neuroscience and Regenerative Medicine, Medical College of Georgia, Augusta University, Augusta, GA, United States; ^2^Department of Psychological Sciences, Augusta University, Augusta, GA, United States

**Keywords:** Bayesian inference, camouflage-breaking, camouflage-learning, cognitive rules of thumb, judgment and decision making, mental shortcuts, visual search

## Abstract

When making decisions under uncertainty, human subjects do not always act as rational decision makers, but often resort to one or more mental “shortcuts”, or heuristics, to arrive at a decision. How do such “top-down” processes affect real-world decisions that must take into account empirical, “bottom-up” sensory evidence? Here we use recognition of camouflaged objects by expert viewers as an exemplar case to demonstrate that the effect of heuristics can be so strong as to override the empirical evidence in favor of heuristic information, even though the latter is random. We provided the viewers a random number that we told them was the estimate of a drone reconnaissance system of the probability that the visual image they were about to see contained a camouflaged target. We then showed them the image. We found that the subjects’ own estimates of the probability of the target in the image reflected the random information they were provided, and ignored the actual evidence in the image. However, when the heuristic information was not provided, the same subjects were highly successful in finding the target in the same set of images, indicating that the effect was solely attributable to the availability of heuristic information. Two additional experiments confirmed that this effect was not idiosyncratic to camouflage images, visual search task, or the subjects’ prior training or expertise. Together, these results demonstrate a novel aspect of the interaction between heuristics and sensory information during real-world decision making, where the former can be strong enough to veto the latter. This ‘heuristic vetoing’ is distinct from the vetoing of sensory information that occurs in certain visual illusions.

## Introduction

A large body of previous research has shown that visual perception can be understood as statistical inference, whereby the brain arrives at a likely interpretation of a given visual scene by jointly evaluating the information it receives from the eyes, what it knows about the visual world, and the potential risks and rewards of a given interpretation (for reviews, see Geisler and Kersten, 2002; Kersten et al., 2004). More generally, studies have shown that statistical (Bayesian) inference provides a useful, quantitative framework of quantitatively understanding the outcome in many sensorimotor tasks. For instance, Bayesian framework can accurately predict the outcomes even on a ‘retail,’ *i.e*., trial-to-trial basis, which makes it useful for the study in many aspects of real-world decision making in which the decisions must be made on a case-by-case basis based on the information about a given case. Indeed, in many cases, the brain functions much like a perfectly rational decision maker, *i.e.*, an Ideal Observer, that combines the various aforementioned probabilistic factors in a computationally optimal fashion (Geisler and Kersten, 2002; Kersten et al., 2004; Geisler, 2011). Remarkably, it turns out that even in case of the phenomena such as visual illusions which, at first blush, might appear to violate the rules of rationality, the perceptual outcome accurately reflects the inferences of a rational decision maker, *i.e*., that of a Bayesian Ideal Observer (Geisler and Kersten, 2002; Kersten et al., 2004; Geisler, 2011).

On the other hand, research has also shown human rationality in decision making has its limits (Tversky and Kahneman, 1974; Kahneman et al., 1982; Simon, 1982; Kahneman, 2013; Thaler, 2015). One influential line of research in bounded rationality, established by Tversky and Kahneman, has shown that human subjects often resort to ‘mental shortcuts’ or heuristics when making judgments and decisions under uncertainty (Tversky and Kahneman, 1974; Kahneman et al., 1982; Kahneman, 2013). The overall motivation for this study was to further elucidate these deviations from Bayesian optimality. More specifically, the present study aimed to characterize the *interaction* between the heuristic factors on the one hand and the effects of other, possibly countervailing factors on the other hand (also see below).

Extensive previous research has established that using heuristics is a natural tendency of the human mind (for overviews, see Gigerenzer and Gaissmaier, 2011; Kahneman, 2013). It is known to occur in naïve subjects as well as highly trained experts (Ericsson, 2018), and has been found in every area of human decision-making examined so far (Gigerenzer and Gaissmaier, 2011; Kahneman, 2013). While the use of heuristics does have its advantages (Kahneman, 2013; Gigerenzer, 2015), the main disadvantage is that judgments (or estimates, in statistical parlance) based on heuristics can result in systematic errors, or biases (Tversky and Kahneman, 1974).

Classical studies of heuristics have typically characterized the decision-making behavior using a paradigm where subjects are presented with vignettes of conceptual or hypothetical problem scenarios and asked to make judgments about the problem (Kahneman, 2013; Raab and Gigerenzer, 2015). For instance, in their classical study of the anchoring and adjustment (AAA) heuristic, Tversky and Kahneman asked two groups of high school students to estimate the product of the sequence of numbers from 1 to 8 within five seconds (Tversky and Kahneman, 1974). One group was presented the descending sequence (8 × 7 × 6 × 5 × 4 × 3 × 2 × 1), and the other group was presented the ascending sequence (1 × 2 × 3 × 4 × 5 × 6 × 7 × 8). The median estimates for the ascending and descending sequences were 512 and 2,250, respectively (the correct answer being 40,320), depending on the group. But decision-making under real-world conditions can be substantially different, in three interrelated respects: First, the decisions cannot be based on the cognitive (or ‘top-down’) information alone, but must take into account ‘bottom-up’ empirical information gleaned from the sensory faculties (Samei and Krupinski, 2010). Second, oftentimes real-world decisions must be made not in the aggregate, but on a case-by-case basis based on information specific to the problem at hand. Third, the observer’s ability to glean and evaluate the sensory information can affect the decisions. However, the role of heuristics during such real-world, “retail” decision-making by experts remains unclear.

To help address this issue, we used recognition of camouflaged objects, or “camouflage-breaking,” by expert observers as an exemplar case. We have previously shown when an object of interest, or target, is effectively camouflaged against its background, naïve, untrained observers cannot recognize the camouflaged target (or “break” its camouflage) (Chen and Hegdé, 2012a,b). However, subjects can be trained in the laboratory to become expert camouflage-breakers (Chen and Hegdé, 2012a). Thus, camouflage-breaking is an excellent model system for studying real-world, retail decision-making by experts. We therefore examined the effects of the AAA heuristic on camouflage-breaking. As described below, we used a straightforward modification of the classical AAA paradigm (Tversky and Kahneman, 1974) to characterize the effects of AAA on visual search for a camouflaged target in a camouflage scene. For this reason, we also present and discuss our results using AAA as the primary framework of understanding.

## Experiment 1: Characterization of the Effect of the Anchoring and Adjustment Heuristic on Camouflage-Breaking in Visual Scenes

### Materials and Methods

#### Subjects

All procedures used in this study were duly reviewed and approved in advance by the Institutional Review Board (IRB) of Augusta University in Augusta, GA, where this study was carried out. All subjects were adult volunteers who had normal or corrected-to-normal vision, and provided written informed consent prior to participating in the study.

**FIGURE 1:**
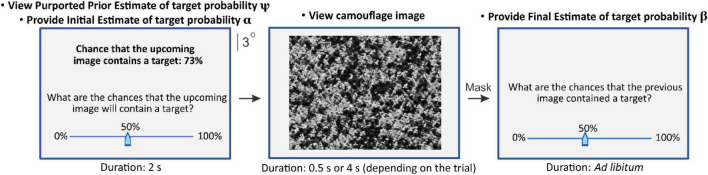
Task paradigm of Experiment 1. The three panels from left to right in this figure are shown in the temporal order they were presented during each trial. The starting position of the on-screen slider (**left** and **right** panels, **bottom**) was always 50%. Panels not drawn to exact scale. See text for details.

Prior to their participation in these experiments, we used our previously described deep-training method (Chen and Hegdé, 2012a) to train the subjects to break camouflage using the same background texture (*e.g.*, foliage, see Figure 1) as the texture they would encounter during the present study (see Chen and Hegdé, 2012a for details). All the subjects who participated in this study had an asymptotic camouflage-breaking performance of *d*′ > = 1.95 (*p* < 0.05) for the background texture that they were to encounter during this study (Chen and Hegdé, 2012a).

Six subjects trained to asymptotic levels participated in Experiment 1.

We digitally synthesized the camouflaged visual scenes used in this study *de novo* as we have previously described (Chen and Hegdé, 2012a). Briefly, each scene consisted of a textured background with or without a single foreground object of interest, *i.e*., the search target. We created background textures that captured key statistical properties of real-world textures using the texture synthesis algorithm of Portilla and Simoncelli, 1999). For instance, to create the background texture type we named “foliage”, we used a real-world photograph of foliage as input, and synthesized a large number of images that captured the key statistical properties of the input texture (see, *e.g*., Figure 1, *center*), so that the output images had the same statistical properties, but were pixelwise non-identical to each other. To create a camouflaged scene with a target for this experiment, we digitally textured a 3-D model of a human face using one of the output images, and composited it, without shadows or occlusion, against a different output image. An equal number of additional output images served as scenes without the target, so that the stimulus during each given trial had a 50% chance of containing a target (see Chen and Hegdé, 2012a for details).

#### Procedure

Prior to the actual data collection, subjects received detailed, illustrated instructions about the trial procedures. Subjects were encouraged to carry out practice trials before starting the actual trials to familiarize themselves with the procedure. The data from the practice trials were discarded.

Experiment 1 consisted of four conditions. During conditions in which explicit anchoring information was externally provided (conditions 1 and 2, Table 1; also see below), each trial began when the subject indicated readiness by pressing a key on the computer’s keyboard, upon which the subject was shown, for 2s, an on-screen message stating the percent chance (which ranged between 0 and 100%, depending on the trial) that the image they were about see contained the search target, *i.e*., a single camouflaged face (Figure 1, *left panel, top*). For convenience, we will refer to this estimate as “purported prior estimate ψ” or, equivalently, “anchoring information”. The subjects were told that this probability was determined by a drone system that reconnoitered the scene for this target. But in actuality, these were pseudorandom numbers generated *de novo* by a random number generator during each trial (also see below).

**TABLE 1:** Experimental conditions in Experiment 1.

Condition #	Anchoring Information	Target status of the image
1	Provided	Target absent
2	Provided	Target present
3	Not provided	Target absent
4	Not provided	Target present

Subjects were then given *ad libitum* time to provide an initial estimate of their perceived probability that the upcoming image contained the search target (“subject’s initial estimate α”) using an on-screen slider (Figure 1, *left panel, bottom*). A previously unseen camouflaged scene was then presented for 0.5 s or 4 s, depending on the trial (Figure 1, *middle panel*), followed by a 0.5 s random-dot mask (not shown). After this, subjects were given *ad libitum* time to estimate the probability that the scene they just viewed contained a target (“subject’s final estimate β”; Figure 1, *right panel*).

The conditions in which no explicit anchoring information was provided (conditions 3 and 4; see Table 1), were identical to conditions 1 and 2, respectively, except that the purported prior estimate was blank (“–“).

Each trial block consisted of eight trials (four conditions × two stimulus durations) presented in a randomly interleaved fashion. Each subject performed at least four blocks of trials over one or more days.

*Rationale for using random numbers for purported prior probabilities* ψ. As noted above, an overall goal of the present study was to characterize the effect of the subjects’ anchoring information ψ on their probability estimates. This meant, on the one hand, that we needed to manipulate ψ. On the other hand, we had to ensure that ψ conveyed no systematic information about the target status of the stimulus, so as to prevent confounding effects. Using random ψ values was a principled way of meeting both of these requirements.

It is important to note that our IRB has determined that our use of random numbers does not amount to deception under the applicable regulations and policies.

#### Data Analysis

Data were analyzed using scripts custom-written for R^1^ and Matlab^2^ platforms. Area under the ROC curve (AUC) was calculated using the default options in the AUC function of the R library *DescTools* (Signorell et al., 2020).

##### *Post hoc* Power Analyses

These analyses were carried out using the R library *pwr*. Before initiating the present study, we carried out *a priori* power analyses to determine the subject recruitment target. To do this, we used the empirically observed fit of the data from a pilot study (Branch et al., 2022) as the expected effect size, and calculated the total number of trials (pooled across all subjects). The results indicated that at least 47 trials (pooled across all subjects and repetitions) would be needed to achieve a statistical power of 0.90. *A posteriori* power analyses using the actual data indicated that our data achieved a power of > 0.95 for the regression analyses in each of the three experiments.

## Results and Discussion

### Effect of the Anchoring and Adjustment Heuristic on Camouflage-Breaking in Visual Scenes: Experiment 1

Prior to participating in this experiment, subjects were trained to criterion in the camouflage-breaking task (mean *d′* = 2.08; median = 1.96; SEM = 0.13) as described in Materials and Methods. The background texture used in this experiment was synthesized from using real-world pictures of natural foliage. The target, when present, was a human head, and was also textured using a different image of the same texture type (*i.e.*, “foliage”). The camouflage images used in this experiment were a random subset of the same large superset of >10^4^ images from which the images used in the training of the subjects were also drawn. That is, the subjects were tested in this experiment using the same type of target and background texture that were used during their prior training.

### Trials Without Anchoring Information

Our task paradigm required the subjects to provide an initial estimate α of the chances that the camouflage image they had not seen yet (but were about to see) contained a target. For convenience, we will refer to this starting estimate of the subjects as their anchored position. When the purported prior estimate ψ was not provided to the subjects during a given trial, the subjects had no explicit information on which to base their initial estimates. For convenience, we will refer to these trials as those in which anchoring information was unavailable or trials without anchoring information.

**FIGURE 2:**
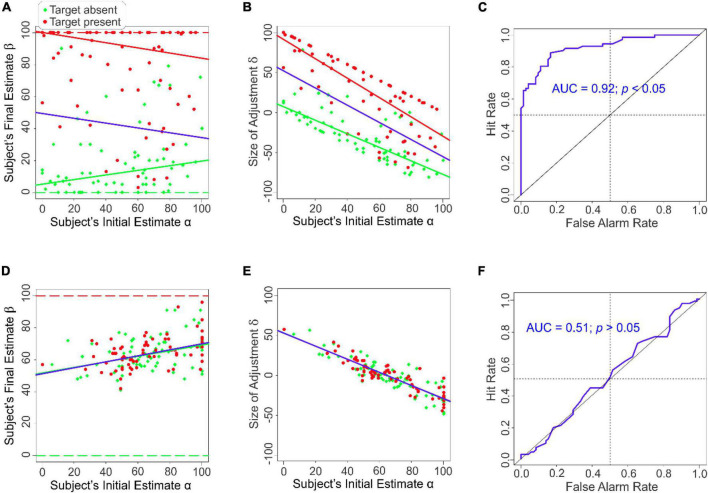
Task performance with or without anchoring information in Experiment 1. Panels **(A–C)** results when the external anchoring information was not provided (*i.e.*, Conditions 3 and 4). Panels **(D,E)** results when the external anchoring information was provided (*i.e*., Conditions 1 and 2). **(A)** Subjects’ final estimates as a function of their initial estimates in the absence of anchoring information. **(B)** The magnitude of the subjects’ adjustment δ as a function of their initial estimate α in the absence of anchoring information. **(C)** ROC analysis of the subjects’ final estimates in the absence of anchoring information. **(D)** The subjects’ final estimates as a function of their initial estimates in the presence of anchoring information. **(E)** The magnitude of the subjects’ adjustment δ as a function of their initial estimate α the presence of anchoring information. **(F)** ROC analysis of the subjects’ final estimates in the presence of anchoring information. Regression lines that best account for the data are shown in a color-coded fashion in panels **(A,B,D,E)** (*red*, target present; *green*, target absent; *blue*, all data points). Note that in panels d and e, the *blue* line largely overlaps, and therefore obscures, the *red* and the *green* lines. The *dashed lines* in panels **(A,D)** denote the expected responses (*red*, target present; *green*, target absent).

As expected, when the anchoring information was unavailable, the subjects tended to estimate the target probability at around 50% on average before they viewed the image (*Subjects’ Initial Estimates*α; *x-axis* in Figure 2A). After viewing the image, the subjects’ final estimates β of target probability were broadly distributed (*y-axis* in Figure 2A), indicating that viewing the image substantially altered their estimates of target probability.

Classical studies have shown that in AAA based on vignettes, subjects start with an initial judgment “anchored” based on the anchoring information, and arrive at their final estimate by adjusting their estimate until they are satisfied with it (Tversky and Kahneman, 1974). The biases, or errors, in these judgments arise from the fact that the subjects’ final judgments tend to be influenced by their initial judgments.

To determine if this also occurs in the absence of anchoring information, we plotted the size of adjustment δ*_*i*_* during a given trial *i* (*i.e*., the amount by which the subjects adjusted their final estimate β_i_ relative to their initial estimate α*_*i*_* during a given trial *i*; δ*_*i*_* = β*_*i*_* −α*_*i*_*) as a function of their initial estimate α*_*i*_* during that trial (Figure 2B). The two quantities were significantly anticorrelated (*r* = −0.57, *df* = 142, *p* < 0.05) indicating that, in this case, the anchored position did contribute to the final estimate even in the absence of the anchoring information. That is, adjustment from an anchored position can occur even in the absence of explicit anchoring information akin to that provided in the classical studies of Tversky and Kahneman (1974). Thus, the anchoring process is dissociable from anchoring information *per se*.

### Subjects Break Camouflage Accurately When the Anchoring Information Is Unavailable

The fact that the AAA effect did occur (albeit on a much smaller scale) when the anchoring information was unavailable raises an important issue: The subjects had to come up with their initial estimates α before they had seen the image for that trial. They provided their final estimates β after they had viewed the stimulus. The fact that β values were significantly correlated with the corresponding α values straightforwardly means that the initial values influenced the subjects’ final estimates. The net effect, if any, of such image-irrelevant factors, by definition, is to degrade camouflage-breaking performance. Were the expert subjects able to overcome the biasing influence of their own initial estimates enough to accurately detect camouflaged targets in the images?

To help answer this question, we carried out a receiver operating characteristic (ROC) analysis of the subjects’ final responses. The resulting ROC curve is shown in Figure 2C (*solid blue line*). The diagonal represents random performance. In this case, the area under the curve (AUC) is 0.5. The actual AUC was significantly above random levels (AUC = 0.92; randomization test, *p* < 0.05, *i.e*., 0 out of 1,000 rounds of randomization). Thus, even though the subjects’ initial positions α did have a biasing effect on their final estimates, the subjects successfully overcame this effect in their final estimates and detected the camouflaged target highly accurately.

To help determine the contributions of various underlying factors to the final estimates γ, we carried out a regression analysis (see “Materials and Methods” section). When the anchoring information was unavailable (Table 2A), the target status θ was a highly significant contributor to the final estimates γ (row 2). Indeed, no other explanatory variable accounted for a significant proportion of the final estimates (rows 1 and 3).

**TABLE 2A:** Contribution of the various explanatory variables to the size of adjustment d when anchoring information was unavailable in Experiment 1 (Conditions 3 and 4).

Row #	Explanatory variable	Estimated coefficient	Standard error	*t* value	*p* value
1	Subjects’ initial estimate α	−0.04	0.08	–0.52	0.60
2	Target status θ (target present *vs*. target absent)	29.32	2.19	13.40	<0.001
3	Reaction time *r*	−0.0003	0.0004	0.69	0.49

### In the Presence of Anchoring Information, the Subjects’ Camouflage-Breaking Performance Is at Random Levels

When the anchoring information, *i.e*., the purported prior estimates ψ, were available, the subjects’ initial estimates α were highly correlated with prior estimates (correlation coefficient *r* = 0.95, *df* = 142, *p* < 0.05; not shown), indicating that the purported prior estimate did succeed in producing a strong anchoring effect as expected. That is, subjects were strongly influenced by this ‘top-down’ information and tended to anchor their own initial estimates on this information. Recall that the purported prior estimates ψ were random.

The subjects were then shown, in a randomized order, the same set of images as those shown when the anchoring information was unavailable. Thus, the differences in outcome between the two pairs of conditions, if any, were not attributable to the images *per se*.

Note that, after viewing the image, the subjects were required to estimate the chance that the image they had just viewed contained a target, and that the sole relevant source of information for estimating this quantity was the image itself. If the subjects solely relied on the image information, their final estimates β would conform to the ground truth about the given image (*red* and *green dashed lines* in Figure 2D). However, the subjects’ actual final estimates of the target status of images substantially varied from the ground truth, regardless of whether the images were positive or negative for the target (*red* and *green symbols* in Figure 2D).

To help characterize the relationship of the magnitude of adjustment δ to the anchored position in the presence of anchoring information, we plotted the size of adjustment δ*_*i*_* during each given trial *i* as a function of their initial estimate α*_*i*_* during that trial (Figure 2E). We found that δ was highly anticorrelated with α, regardless of the target status θ of the image (*r* = −0.89, *df* = 142, *p* < 0.05; Figure 2E). This straightforwardly suggests that the reason why the final estimates were *uncorrelated* with the target status θ of the image (Figure 2D) was that the subjects arrived at their final estimates β by adjusting from their anchored positions α (Figure 2E), which themselves were highly correlated with the random ψ values (*r* = 0.53, *df* = 142, *p* < 0.05; not shown).

*Post hoc* modeling of the subjects’ final estimates confirmed that the actual target status of the image indeed played an insignificant role in the subjects’ final estimates of the target (Table 2B, row 2). Indeed, the only predictor that significantly accounted for the final estimates were the subjects’ initial estimates α (row 1). Receiver operating characteristic (ROC) analysis indicated that subjects’ performance was indistinguishable from random (Figure 2F). Note that this effect is not attributable to the subjects’ intrinsic inability to break camouflage to begin with, because when the anchoring information was unavailable, the same subjects broke camouflage highly accurately using the same set of images.

**TABLE 2B:** Contribution of the various explanatory variables to the size of adjustment d when anchoring information was available in Experiment 1 (Conditions 1 and 2).

Row #	Explanatory variable	Estimated coefficient	Standard error	*t* value	*p* value
1	Subjects’ initial estimate α	0.19	0.04	5.43	<0.001
2	Target status θ (target present *vs*. target absent)	0.20	0.79	0.25	0.80
3	Reaction time *r*	0.0002	0.0003	0.80	0.43

The result that the subjects performed at random levels is consistent with the fact that the anchoring information ψ that their decisions were based on was itself random. This result is nonetheless surprising, because it suggests that trained subjects can altogether ignore task-relevant empirical information in camouflage scenes when they have access to anchoring information. One plausible explanation for this is that the subjects were under time pressure so that they were unable to scrutinize the images sufficiently well. Previous studies have shown that time pressure can induce subjects to resort to using heuristics (Kahneman et al., 1982; Kahneman, 2013). However, our *post hoc* analyses indicated that the stimulus duration did not significantly contribute to the outcome, regardless of the target status (row 3, Tables 2A,B). Moreover, subjects often took less than the allotted time before responding (data not shown; also see Experiment 2 below).

## Experiment 2: Does the Effect of Anchoring and Adjustment Generalize to Other Experimental Conditions?

### Materials and Methods

#### Subjects

Four subjects trained to asymptotic levels participated in Experiment 2.

#### Procedure

**FIGURE 3:**
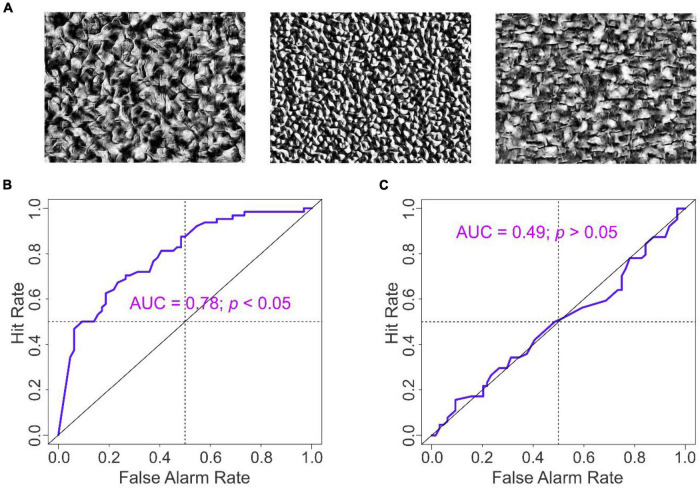
Results of Experiment 2. **(A)** exemplar stimuli used in Experiment 2. **(B,C)** ROC analysis of the subjects’ final estimates in the absence and presence of anchoring information, respectively.

This experiment was identical to Experiment 1, except in the following three respects. First, three new background textures (“fruit,” “nuts,” and “mushrooms”; see Figure 3A; also see Table 3) were used as background textures, and counter-rotated across trials, blocks, and subjects. Second, novel, naturalistic 3-D objects, called “digital embryos” that the subjects had not seen before were used as targets in 50% of randomly interleaved trials, also on a counter-rotating basis (not shown). Third, the subjects were allowed to view the stimuli for an unlimited duration and were allowed to end the stimulus presentation and proceed to the next phase of the trial by pressing a designated button (not shown).

**TABLE 3:** Experimental conditions in Experiment 2.

Condition #	Anchoring Information	Target status of the image
1	Provided	Target absent
2	Provided	Target present
3	Not provided	Target absent
4	Not provided	Target present

## Results and Discussion

### Anchoring and Adjustment Effects Are Reproducible Across Disparate Experimental Conditions

To determine whether and to what extent the AAA effect generalizes across to other experimental parameters, we carried out Experiment 2, in which we systematically varied the background texture and the search targets (see “Materials and Methods” section for details; also see Figure 3A).

We found that all of the key results of Experiment 1 were reproducible in this experiment as well (Figures 3B,C). For instance, when the purported prior estimates ψ were available, the magnitude of adjustment δ was strongly anticorrelated with α regardless of the target status θ of the image when the anchoring information was available (*r* = −0.79, *df* = 126, *p* < 0.05; not shown). When the prior information was unavailable, the anticorrelation between δ and α was weaker, albeit still statistically significant (*r* = −0.44, *df* = 126, *p* < 0.05; not shown). Finally, the subjects’ camouflage-breaking performance was highly accurate when anchoring information was unavailable (AUC = 0.78, *p* < 0.05), but was at random levels when anchoring information was available (AUC = 0.49, *p* > 0.05). The results of the regression analyses for this experiment (Tables 4A,B) were qualitatively similar to those from Experiment 1. Thus, the results of Experiment 1 were essentially reproducible in Experiment 2.

**TABLE 4A:** Contribution of the various explanatory variables to the final estimates γ when anchoring information was available in Experiment 2 (Conditions 3 and 4): *Post hoc* general linear modeling (GLM) of the contributions of the various explanatory variables to the response variable (*i.e*., final estimates γ of subjects).

Row #	Explanatory variable	Estimated coefficient	Standard error	*t* value	*p* value
1	Subjects’ initial estimate α	0.04	0.15	0.26	0.80
2	Target status θ (target present *vs*. target absent)	9.39	1.78	5.27	<0.001
3	Reaction time *r*	0.0005	0.007	0.07	0.94
					

**TABLE 4B:** Contribution of the various explanatory variables to the final estimates γ when anchoring information was available in Experiment 2 (Conditions 1 and 2): *Post hoc* general linear modeling (GLM) of the contributions of the various explanatory variables to the response variable (*i.e*., final estimates γ of subjects).

Row #	Explanatory variable	Estimated coefficient	Standard error	*t* value	*p* value
1	Subjects’ initial estimate α	0.04	0.07	0.55	0.58
2	Target status θ (target present *vs*. target absent)	−0.11	1.07	–0.11	0.92
3	Reaction time *r*	−0.01	0.006	–1.70	0.09

## Experiment 3: Visual Pattern Detection Performance of Naïve, Non-Professional Subjects With *Vs.* Without Anchoring Information

### Materials and Methods

#### Subjects

Eleven naïve, non-professional subjects (as opposed to trained camouflage-breakers used in Experiments 1 and 2) participated in Experiment 3.

#### Procedure

**FIGURE 4:**
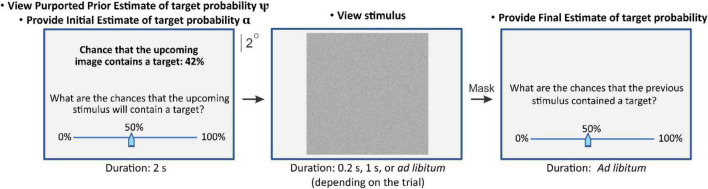
Task paradigm of Experiment 3. In this experiment, the visual stimulus was a dynamic random dot stimulus (dRDS), one static frame of which is shown in this figure (middle panel). In 50% of the randomly interleaved trials, the dRDS contained in Gabor patch at the subject’s contrast threshold (Kersten, 1984). See text for details.

This experiment was identical to Experiments 1 and 2, except where specified otherwise. The subjects performed a target detection task as in Experiments 1 and 2, except that the target in this experiment was a Gabor patch (8 cycles/degree, σ = 1^°^) embedded in dynamic random dot noise (Kersten, 1984) (dot density, dot size = 1 pixel^2^; 50% ON, 50% OFF; refresh rate = 60 Hz; see Figure 4). Prior to the experiment, subjects received detailed instructions and viewed exemplar images with or without Gabor patches (clearly discernible when present), so that subjects knew what to look for. Collectively, these procedures helped ensure that no prior training or visual pattern recognition expertise was needed in order for the subjects to perform the task (see Table 5). To help add stimulus uncertainty, the spatial location and orientation of the Gabor patch (when present) were randomly jittered from one trial to the next.

**TABLE 5:** Experimental conditions in Experiment 3.

Condition #	Anchoring Information	Target status of the stimulus
1	Provided	Gabor patch absent
2	Provided	Gabor patch present
3	Not provided	Gabor patch absent
4	Not provided	Gabor patch present

We customized the contrast of the Gabor patch for each subject, so as to help ensure that the stimulus was sufficiently ambiguous and to help minimize the variations in task performance related to task difficulty across subjects. We carried out a preliminary experiment to determine the contrast threshold for each subject. To do this, we presented the Gabor patch (with the same parameters as above), one per trial at systematically varying contrasts. Subjects viewed the stimulus *ad libitum*, followed by a random dot mask, and used an on-screen slider to report the probability that the stimulus contained the Gabor patch target. We fitted a logistic contrast response function (Harvey, 1997) to the data (Supplementary Figure 1A). We took the point of inflection of the fitted function, at which the slope of the function was maximal, as the contrast threshold for the given subject (Campbell and Green, 1965). The distribution of contrast thresholds for all subjects is shown in Supplementary Figure 1B.

For each subject, the Gabor patch target in Experiment 3 was presented at their contrast threshold. The subject performed the target detection as in Experiments 1 and 2, except that the target was the Gabor patch, instead of a camouflaged target.

## Results and Discussion

### Anchoring and Adjustment Effects Are Reproducible in Naïve, Untrained Subjects Performing a Simple Detection Task

To determine if this overriding effect of AAA is specific to experts such as highly trained camouflage-breakers, we tested naïve, non-professional subjects using a variation of the above task that required neither training nor expertise in pattern recognition (Experiment 3; see “Materials and Methods” section for details). This experiment was identical to Experiments 1 and 2, except that the subjects were required to report whether a dynamic random dot stimulus contained a Gabor patch presented at the subject’s empirically determined contrast threshold (see Figure 4; also see Supplementary Figure 1). The subjects were told that the prior information provided to them was the probability that the image they were about to see did contain the Gabor target, as determined by a previous viewer.

**FIGURE 5:**
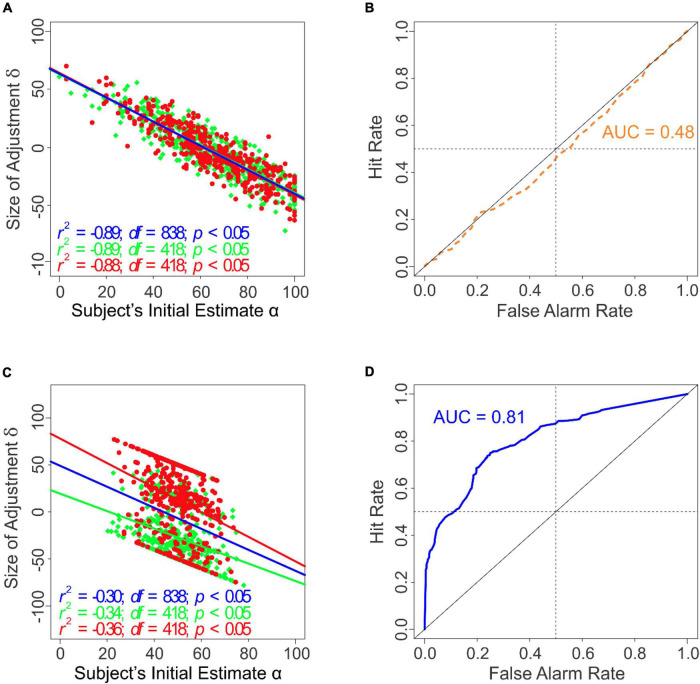
Task performance of subjects with or without anchoring information in Experiment 3. The various panels in this figure are drawn using the same plotting conventions as the corresponding panels in earlier figures. **(A)** The magnitude of the subjects’ adjustment δ as a function of their initial estimate α in the absence of anchoring information. Note that the *blue* regression line in this panel largely overlaps, and therefore obscures, the *red* and the *green* regression lines. **(B)** ROC analysis of the subjects’ final estimates in the presence of anchoring information. **(C)** The magnitude of the subjects’ adjustment δ as a function of their initial estimate α in the absence of anchoring information. **(D)** ROC analysis of the subjects’ final estimates in the absence of anchoring information.

**FIGURE 6:**
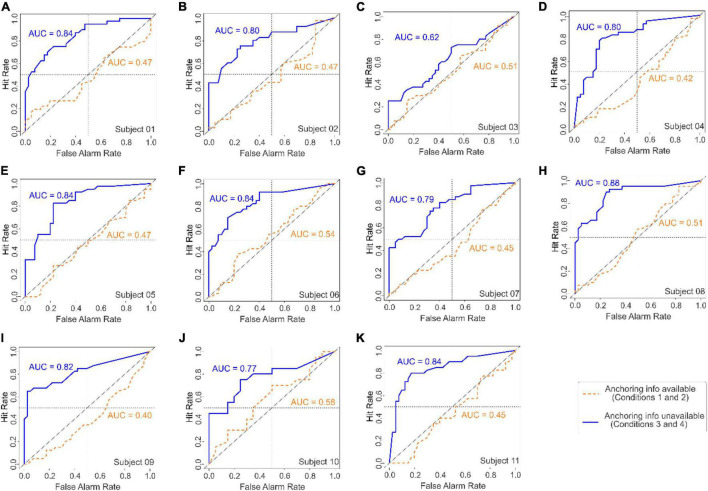
ROC analyses of the responses of each of the 11 individual subjects in Experiment 3 (panels **A-K**). In each panel, the ROC curves for Gabor detection performances with or without anchoring information (dashed *brown* and *solid blue curves*, respectively) are shown, as are the corresponding AUC values (*brown* and *blue* type, respectively). In each panel, the *diagonal* represents chance performance (AUC = 0.5). See text for details.

The results of this experiment (Figure 5) were qualitatively similar to those of Experiments 1 and 2 (Figures 2,3, respectively). Moreover, each individual subject in Experiment 3 detected the target accurately in the absence of the anchoring information, but performed at chance levels in the presence of anchoring information (Figure 6). Thus, the ability of the AAA heuristic to override the empirical information generalized across stimuli, tasks, and the subject’s training/expertise in pattern recognition.

Two additional aspects of Experiments 1-3 are worth noting and are clearest from the results of Experiment 3. First, the subjects’ use of the AAA heuristic is not attributable to time pressure *per se*, because the subjects performed highly accurately under otherwise identical conditions when anchoring information was not available (Figures 3, 5). Second, the anchoring effects in this experiment were not attributable to the *requirement* to report the initial estimate *per se*, because the subjects were required to make this report regardless of whether anchoring information was present (Tables 6A,B). When the anchoring information ψ was available, the amount of adjustment δ was highly anticorrelated with the initial values α (*r* = −0.89; *df* = 838; *p* < 0.05; Figure 5A), and was not significantly influenced by the presence of the Gabor patch θ (1-way ANCOVA; α: *F*(1,836) = 3088.47, *p* < 2.0 × 10^–16^; θ: *F*(2,836) = 0.973, *p* = 0.32). When the anchoring information was unavailable, the anticorrelation was more modest, albeit still significant (*r* = −0.30; *df* = 838; *p* < 0.05; Figure 5C), arguably because the subjects took into account the presence of the Gabor patch θ when the anchoring information α was unavailable (1-way ANCOVA; α: *F*(1,836) = 118.13, *p* < 2 × 10^–16^; θ: *F*(2,836) = 351.99, *p* < 2 × 10^–16^). Thus, the anchoring process itself is dissociable from the anchoring information it is based on, in that the former can occur without the latter.

**TABLE 6A:** Contribution of the various explanatory variables to the final estimates γ when anchoring information was available in Experiment 3 (Conditions 1 and 2): *Post hoc* general linear modeling (GLM) of the contributions of the various explanatory variables to the response variable (*i.e*., final estimates γ of the subjects).

Row #	Explanatory variable	Estimated coefficient	Standard error	*t* value	*p* value
1	Subjects’ initial estimate α	0.48	0.11	4.40	<0.001
2	Target status θ (target present *vs*. target absent)	−0.69	0.75	–0.92	0.36
3	Reaction time *r*	3.01 × 10^–3^	2.47 × 10^–3^	1.22	0.22
					

**TABLE 6B:** Contribution of the various explanatory variables to the final estimates γ when anchoring information was available in Experiment 3 (Conditions 3 and 4): *Post hoc* general linear modeling (GLM) of the contributions of the various explanatory variables to the response variable (*i.e*., final estimates γ of the subjects).

Row #	Explanatory variable	Estimated coefficient	Standard error	*t* value	*p* value
1	Subjects’ initial estimate α	–1.10	0.10	–10.65	<0.001
2	Target status θ (target present *vs*. target absent)	38.98	2.08	18.72	<0.001
3	Reaction time *r*	5.67 × 10^–3^	6.62 × 10^–3^	0.86	0.39

## General Discussion

### A New Principle of Top-Down *vs*. Bottom-Up Interaction: Anchoring and Adjustment Heuristic Can ‘Veto’ Visual Information

We show that, in each of the three experiments, the subjects fail to detect the target when anchoring information is available. But when anchoring information is unavailable, the same subjects detect the target highly accurately using the same set of images. This straightforwardly implies that the anchoring information causes the subjects to ignore the image information in favor of the anchoring information when the latter is available. That is, the heuristic information can override or veto the image information in visual pattern recognition tasks.

Our results demonstrate that there are certain conditions, such as the availability of strong anchoring information in the present case, under which heuristic decision-making is the default mode, and not the strategy of last resort, of decision-making under uncertainty. This is because when both sets of information were available, the subjects’ decisions were dominated by the heuristic information. This finding is particularly important, because the resulting errors were large enough to reduce the subjects’ camouflage-breaking performance to chance levels.

Another notable aspect of our results also show that the biasing effects of AAA, previously demonstrated in the aggregate for subject groups evaluating verbal vignettes (Tversky and Kahneman, 1974; Kahneman et al., 1982; Thaler, 1993; Rieskamp and Hoffrage, 2008), persist in ‘retail’, case-by-case decision-making. Case-by-case decision scenarios are common in the real world, so that the heuristic influences demonstrated by our study are likely to be prevalent under real-world conditions.

Our results also show that the anchoring can occur, albeit to a lesser extent, in the absence of externally provided anchoring information. That it is, even when no anchoring information is externally provided, the subjects’ final estimates are anticorrelated, albeit modestly, with their initial estimates, suggesting that the subjects start from an anchored position even when not induced to do so by externally provided information (see Figures 2B, 5C). It is plausible that the process of providing the initial estimates itself had the implicit effect of anchoring the subjects’ initial judgments. In any event, this internal anchoring was not strong enough to significantly affect the subjects’ performance (see Figures 2C, 5D). More significantly, this effect demonstrates that the anchoring process is dissociable from the anchoring information *per se*. This is important, because this suggests that requiring subjects to make an initial decision can affect their final decision in any task.

Our results raise the possibility that the AAA heuristic can, in principle, affect any task involving visual search. This has serious implications for real-world tasks involving visual search, such as airport baggage screening and medical image perception. Indeed, we have recently found a similar AAA ‘veto’ effect in practicing radiologists examining mammograms (Branch et al., 2022).

### Why Disbelieve Your Own Eyes?

A striking aspect of our results is the fact that subjects effectively disbelieve their own eyes in favor of what they hear from an external source, such as a drone or a previous viewer. In all three experiments, subjects accurately detected the target in the absence of prior information, indicating that the subjects were able to detect the target to begin with, but when the prior information was available, they essentially ignored what they saw in favor what they were told.

The veto effect is all the more striking in the cases of Experiments 1 and 2, where the subjects were expert camouflage-breakers. We have previously reported that expert camouflage-breakers are so skilled in their task that they can detect the camouflaged target even after brief viewing the stimulus, even as briefly as 50 ms, which does not permit extended scrutiny or eye movements (Chen and Hegdé, 2012a; Branch et al., 2021). In this specific sense, detecting the target is relatively easy for the expert subjects, so that the subjects could easily cross-check the prior information against the visual evidence. It is therefore surprising that the subjects – judging by the results – fail to, or choose not to, do such cross-checking. A detailed examination of the cognitive costs of such cross-checking, including the costs imposed by task difficulty, are needed to help clarify the reasons behind this surprising effect.

To be sure, what is surprising here is that the heuristic effect can be so strong, and not that expert camouflage-breakers resort to heuristic decision-making in the first place. After all, heuristic decision-making is notoriously resistant to expertise training; experts in every profession examined to date are known to resort to heuristic decision-making (Gigerenzer and Gaissmaier, 2011; Kahneman, 2013; Ericsson, 2018). But previous studies have neither systematically examined the interaction between the heuristic information versus the sensory evidence. Our study examined this effect and found the veto effect.

Still, why does the veto occur at all? Why do subjects ignore the physical evidence in the images? While our study did not examine this important question for practical reasons, one plausible explanation is that the veto itself is, at least in part, a reflection of the so-called authority bias or halo effect, whereby experts and laypeople alike abide by what they consider expert opinions (Milgram, 1963; Stasiuk et al., 2016; Zaleskiewicz and Gasiorowska, 2021). This may also explain, at least in part, why the subjects apparently do not begin to disregard the prior information even upon a relatively large number of trials in which the prior information does not jive with the empirical evidence before the subjects’ very eyes. The present study did not examine this important issue for practical reasons, in part because it would require, among other things, a detailed quantification of both the perceived reliability of the prior information during a given trial, and the updating of the perceived reliability from one trial to the next. Further studies are needed to examine these important issues in detail.

### Possible Limitations of Heuristic Vetoing and Other Caveats

It is important to emphasize that what our results demonstrate is that under certain conditions, e.g., when the heuristic information is strong and the bottom-up information is ambiguous or otherwise weak, the heuristic information *can* override the visual information. This is not to say, however, that heuristic information always *does* override visual information. The uncertainty of the visual information in our experiments was arguably high enough, *i.e*., the sensory information was weak enough, that the strong top-down information was able to override it.

It is intuitively obvious, on the other hand, that there exist conditions where the opposite is true, *i.e*., the bottom-up information overrides the top-down information. For instance, if the visual targets in our experiments were easily detectable, *e.g.*, if the Weber contrast of the Gabor patches in Experiment 3 were 1.0 and that of the background were 0.0, subjects would readily ignore the prior information and go with the image information instead. For practical reasons, the present study did not examine this possibility. Further studies are needed to empirically establish this possibility.

It is also intuitively obvious that under most real-world conditions, the strength of the stimulus information would be somewhere between the aforementioned two extremes. While the vetoing effect would be obscured in such cases, the underlying heuristic-visual interaction is unlikely to disappear altogether. Instead, the behavioral outcomes under these conditions are likely to reflect a complex interplay of the two influences, when both are present.

### Heuristic-Visual Interaction Is Distinct From Visual Illusions

It is instructive to compare and contrast heuristic vetoing with certain visual illusions. For instance, in the hollow face illusion or the Ames room illusion, the brain’s built-in assumptions about the relevant visual objects override the visual information (Geisler and Kersten, 2002; Hartung et al., 2005; Kroliczak et al., 2006; Parpart et al., 2018). These visual illusions are analogous to the heuristic vetoing, in two main respects. First, in both cases, image information is overshadowed by top-down factors. Second, both represent special cases, where the image information is ambiguous, usually in highly specific ways. For example, the Ames room has to be constructed in specific ways to facilitate the brain’s tendency to assume the room is symmetrical. In the case of heuristic vetoing, the visual target presumably must be difficult enough to find for the vetoing effect to show through. Thus, visual illusions are special cases just as heuristic vetoing is.

On the other hand, heuristic vetoing is distinctly different, in the sense that it is clearly not built-in, but externally induced. In the present case, for instance, the anchoring effect is induced by the anchoring information provided to the subject. The built-in assumptions in the aforementioned visual illusions are typically so strong that it is not possible generally to volitionally alter these influences.

### Concluding Remarks: Heuristic Vetoing in Perspective

Given the aforementioned fact that heuristic vetoing is self-evidently a rather special case in the vein of visual illusions, one reasonable perspective about our study is that it is a proof-of-principle study that reveals that heuristics can, in principle, veto the visual evidence. Also, given the fact that heuristics are ubiquitous in human judgments, what is ultimately surprising about our results is not that they reveal a heuristic effect, but that they reveal a veto effect.

## Data Availability Statement

The data supporting the conclusions of this article will be made available by the authors upon reasonable request.

## Ethics Statement

The studies involving human participants were reviewed and approved by Institutional Review Board (IRB) of Augusta University, Augusta, GA, United States. The participants gave written informed consent prior to participating in the study.

## Author Contributions

FB, EP, and JH designed the experiment, analyzed the data and prepared the manuscript. FB and EP collected the data. All authors contributed to the article and approved the submitted version.

## Conflict of Interest

The authors declare that the research was conducted in the absence of any commercial or financial relationships that could be construed as a potential conflict of interest.

## Publisher’s Note

All claims expressed in this article are solely those of the authors and do not necessarily represent those of their affiliated organizations, or those of the publisher, the editors and the reviewers. Any product that may be evaluated in this article, or claim that may be made by its manufacturer, is not guaranteed or endorsed by the publisher.

## Footnotes

1
r-project.org

2
Mathworks.com
