# Supplementary material for: Heuristic Vetoing: Top-Down Influences of the Anchoring-and-Adjustment Heuristic Can Override the Bottom-Up Information in Visual Images
Source: Front Neurosci. 2022 May 20;16:745269. doi: 10.3389/fnins.2022.745269 (PMC9163416; doi:10.3389/fnins.2022.745269)
Supplement: Supplementary file 1 [file Image_1.pdf]

SUPPLEMENTARY FIGURES

Heuristic Vetoing: Top-Down Influences of the Anchoring-and-Adjustment Heuristic Can Override the Bottom-Up Information in Visual Images

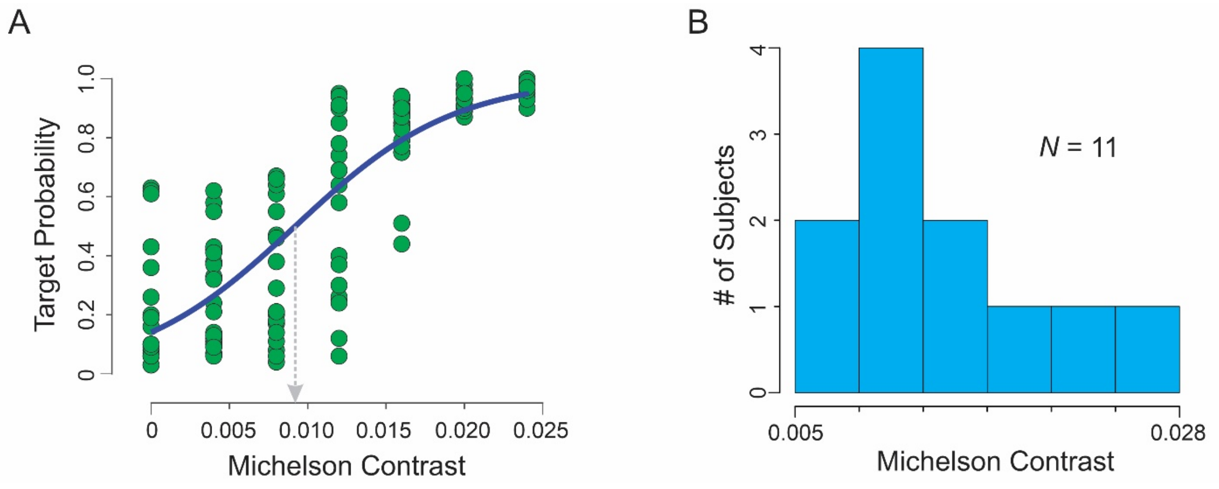

Supplementary Figure S1. Determining the contrast thresholds of individual subjects for Experiment 3. **(A)** Determining the contrast threshold for an exemplar subject. The background consisted of dynamic random dot stimuli (dRDS). The background remained at a constant contrast during each trial, and across all trials and all subjects, but the contrast of the embedded target (if present) varied depending on the trial. The dRDS had an embedded dRDS Gabor patch target in a randomly interleaved 50% of the trials. Gabor patches, when present, were presented at one of the seven contrasts (*x*-axis) depending on the randomly interleaved trial. During each trial, the subject reported the probability that a given dRDS contained the Gabor target (*filled green circles*). The *blue curve* denotes the best-fitting logistic function. The contrast threshold of the subject was defined as the point of inflection, *i.e.*, the point of maximum slope (*dashed vertical line*) of the logistic function**.** **(B)** Distribution of the empirically determined contrast threshold for all 11 subjects in Experiment 3. For each subject, the Gabor targets in Experiment 3 were presented at their empirically determined contrast threshold. See text for details.

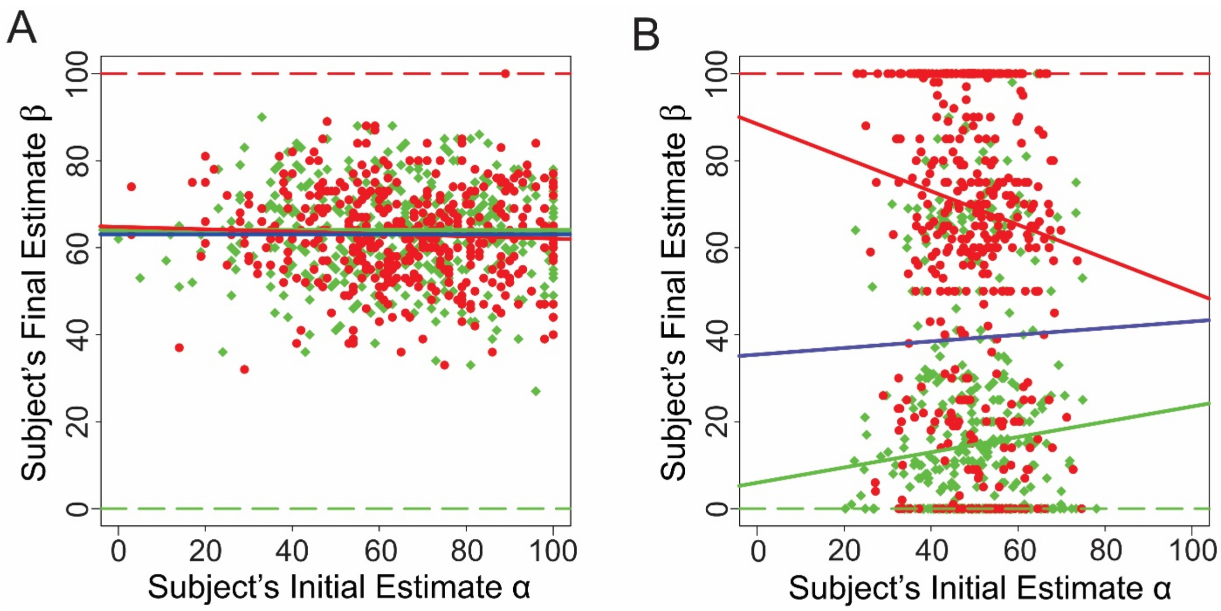

Supplementary Figure S2. Results of Experiment 3. Subjects' reported final estimate β as a function of their initial estimate α with anchoring information (panel **A**) and without anchoring information (panel **B**). See the main text for details. Plotting conventions are as in Fig. 2. Note that in panel a, the *blue* regression line largely overlaps, and therefore obscures, the *red* and the *green* regression lines.
